# Hydatid Cyst Masquerading as Chronic Sialadenitis-an Extremely Rare Locale of Zoonotic Disease Demystified by Cytology

**DOI:** 10.22038/ijorl.2025.81795.3829

**Published:** 2025

**Authors:** Kavita Gaur, Kiran Agarwal, Gautam Bir Singh, Poornima Kumar, Arun Krishna

**Affiliations:** 1 *Assistant Professor, Lady Hardinge Medical College, Shaheed Bhagat Singh Marg, New Delhi, India.*; 2 *Department of Otorhinolaryngology & Head-Neck Surgery, Lady Hardinge Medical college, Shaheed Bhagat Singh Marg, New Delhi, India.*

**Keywords:** Hydatid cyst, Submandibular gland, Cytology

## Abstract

**Introduction:**

Hydatid cyst disguised as chronic sialadenitis, represents a highly unusual clinical presentation. Only rarely do hydatid embryos escape the hepatic and pulmonary vasculature to enter other organ territories. This report highlights a tropical infection hoodwinking clinical suspicion due to an unexpected rare anatomical site of appearance. The present case masqueraded as a comparatively innocuous chronic sialadenitis. In addition, previous work has debated the use of cytology in diagnosing hydatid cyst on the grounds of triggering anaphylaxis.If done carefully, however, rewarding diagnostic returns can be seen, as seen herein.

**Case Report:**

We present an extremely rare case of a 35- year -old female presenting with swelling in the submandibular region with pain for two months. Ultrasonography revealed a hypoechoic lesion with fine needle aspiration showing the presence of numerous refractile hooklets suggesting hydatid disease. The same was confirmed both by serological evaluation and subsequent histopathological findings.

**Conclusion:**

This report highlights a unique presentation of hydatid cyst presenting as chronic sialadenitis, hitherto unreported in medical literature. Furthermore, it documents a safe diagnostic course employing cytology to diagnose atypical echinococcal infections.

## Introduction

 Hydatid cyst is a zoonotic disease and is recognized as a health problem of global prominence. It is endemic in sheep rearing countries such as Australia, New Zealand, Middle East as well as Latin America & the Far East (1). These cysts are mostly seen in the liver and lungs. The submandibular gland is a very rare site for this lesion (2-4). With this background, we present a case of hydatid cyst in the submandibular gland, wherein the clinical presentation was found to be interesting and hitherto unreported in the medical literature. In addition, this report also highlights the importance of fine needle aspiration cytology (FNAC) in the diagnosis of parasitic infections with rare site predilection.

## Case Report

**Fig. 1a:**
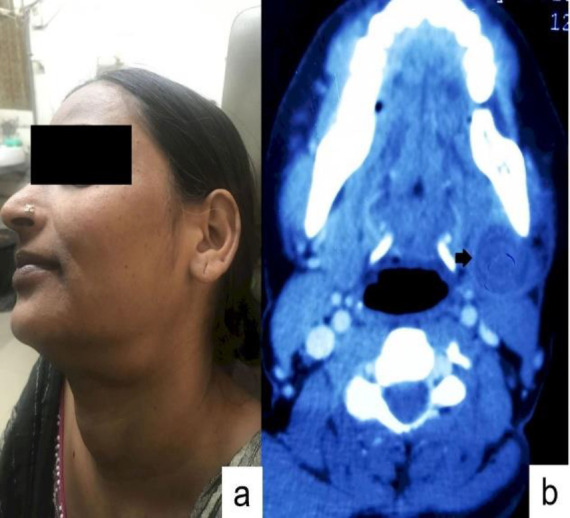
Clinical photograph of the patient showing a submandibular swelling. **b: **CT scan image [axial cut] delineating the hydatid cyst

A 35-year-old female reported to the ENT out-patient clinic with complaints of swelling in the left submandibular region for one month with clinical symptoms suggestive of sialadenitis. The patient had pain in the submandibular region along with submandibular swelling while chewing food for the past two months and the swelling persisted. On examination a well-defined swelling measuring 3 cm in greatest dimension was seen in the left submandibular region (Fig 1a). 

**Fig. 2:**
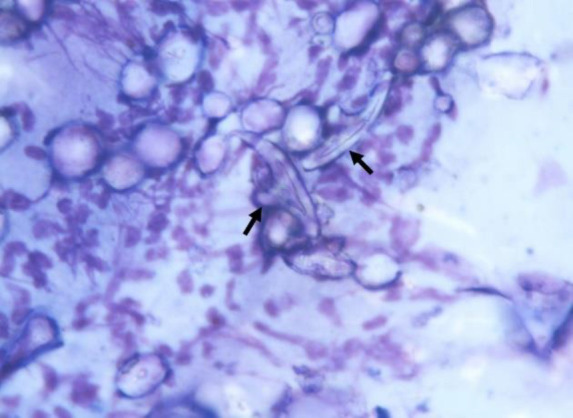
**FNAC:**Giemsa-stained cytosmears show numerous refractile hooklets [Giemsa,100X]

The swelling was firm in consistency and non-tender. It would be prudent to note that the swelling was bimanually palpable. The patient was further investigated on the lines of chronic sialadenitis. Fine needle aspiration cytology (FNAC) and USG neck was done along with x-rays to rule out stones. USG revealed a hypoechoic lesion in the left submandibular gland 30 X 25mm in dimensions. On cytology, aspirate smears showed the presence of refractile hooklets (Figure 2). X-ray occlusal film for submandibular gland was negative for calculi. 

Hence, a presumptive diagnosis of hydatid cyst in the left submandibular gland was made. The patient underwent serological testing for hydatid cyst which was positive for Echinococcus. A CT scan was also done, which revealed a 23 X 22mm well defined rounded cystic lesion with an enhancing wall within the substance of the submandibular gland with separations and calcification within (Fig 1b). The patient was screened for other cystic hydatid lesions by X-ray chest and USG abdomen. 

No other lesion was seen. In accordance with the WHO guidelines for hydatid cyst management, excision of the submandibular gland with follow-up (to detect any recurrent lesion) was planned (2,5). Post-operative recovery was uneventful and the patient was discharged subsequently. 

**Fig. 3:**
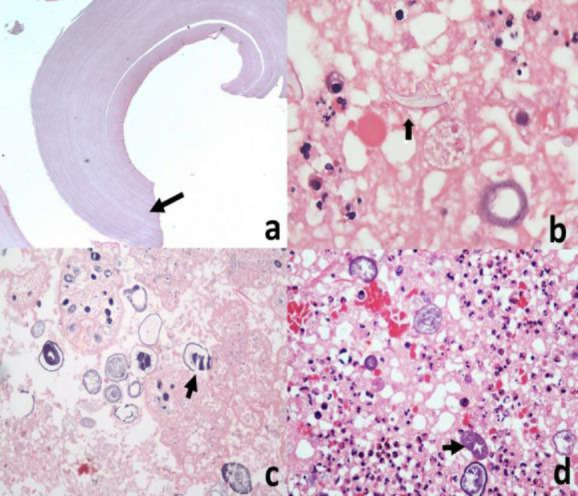
H & E-stained photomicrographs showing a) acellular laminated membrane (black arrow) [H & E, 40X] b) A detached hooklet highlighted by the arrow [ H & E,40X] c) Numerous calcified eggs (black arrow) H & E,100X] d) Calcified eggs, occasional scolex in a necrotic, inflamed background [H & E,100X]

Histopathological analysis showed an ectocyst with numerous brood capsules with scolices. Endogenous daughter cysts with calcification, scolices with suckers and hooklets were also seen. The cyst wall showed plasma cells, histiocytes and eosinophils within the fibrous layer representing pericyst, consistent with hydatid cyst (Figure 3a-d). A one-year follow-up was maintained with no untoward incident to report.

## Discussion

Hydatid cyst is caused by* Echinococcus Granulosus *& rarely by* Echinococcus Multilocularis. *Dogs are the primary hosts and cows, sheep, horses, pigs and human beings are intermediate hosts. The parasitic eggs open up in the small intestines of intermediate hosts (i.e. humans) and reach the liver and lungs through portal venous or lymphatic systems; then forming the hydatid cyst lesions. The embryos may pass over the hepatic sinusoids or pulmonary capillary barriers and rarely enter the systemic circulation to lodge within other organs of the body. Thus, hydatid cysts are predominantly seen in the lungs and the liver and only very rarely in the submandibular gland (1-4,6).

Most of these cysts are asymptomatic. Clinical manifestations of the hydatid cysts are due to the mechanical effects on the involved organ and allergic reaction of the cystic fluid (7). This case presented to us with clinical features suggestive of chronic sialadenitis probably due to the blockage of the ductal system of the gland by the hydatid cyst. 

As the submandibular location is usually not associated with hydatid cysts, the clinical approach was in line with a simple cystic lesion and hence an FNAC was performed. Subsequently, serology and imaging corroborated the diagnosis. CT scan further substantiated our diagnosis and confirmed the site. FNAC is conventionally contraindicated in suspected cases of hydatid cyst because of the risk of anaphylaxis and dissemination (1,3). However, we believe that this risk is over-emphasized, as there are many reports on the cytological diagnosis of hydatid cysts without any complications (7-9). 

We too recorded no urticarial or anaphylactic reaction in our case. A possible pitfall to cytological diagnosis is the aspiration of a hypocellular aspirate with only occasional hooklets/scolices being observed. This may hence be easily overlooked, more so in the absence of clinical/imaging suspicion. 

A judicious employment of centrifugation as performed herein or alternatively cell block methods may improve cellular yield and in turn diagnostic success (10). FNAC along with CT scan helped us to reach to a presumptive clinical diagnosis and promulgate a judicious treatment protocol (whole body screening for additional cyst) and a better surgical approach to avoid spillage of lesion. Moreover, all such cases require long term follow-up to detect any recurrence of the said lesion. In the above context, it would also be pertinent to note that we had a positive serological test for hydatid cyst in the submandibular gland. 

## Conclusion

This case merits mention on many accounts. Firstly, for the rarity of the lesion in the submandibular gland and the gross under reporting of such cases which limits conclusions to be drawn on the clinical course, management and prognosis of the said lesion. Secondly, the clinical presentation in the form of chronic sialadenitis is also unique and finds no mention in literature. 

Lastly, this clinical record also highlights the importance of FNAC in the diagnosis of this lesion in rare sites, emphasizing the additional diagnostic and therapeutic planning required for earnest management. Though the safety of FNA cannot be confirmed without clinical trials or systematic reviews the unremarkable post procedure course as seen herein merits documentation. To conclude hydatid cyst should be kept as an important differential for submandibular gland swellings, albeit a rare one. 
